# The comparison of serum bone-turnover markers in different stage of chronic kidney disease and the associated impact of intradialytic cycling in patients with end-stage renal disease

**DOI:** 10.18632/aging.206177

**Published:** 2025-01-14

**Authors:** Yi-Chou Hou, Chia-Ter Chao, Li-Jane Shih, Kuo-Wang Tsai, Shyh-Min Lin, Ruei-Ming Chen, Kuo-Cheng Lu

**Affiliations:** 1 Department of Internal Medicine, Division of Nephrology, Cardinal Tien Hospital, School of Medicine, Fu Jen Catholic University, New Taipei City, Taiwan; 2 Department of Internal Medicine, Nephrology division, National Taiwan University Hospital, National Taiwan University College of Medicine, Taipei, Taiwan; 3 Graduate Institute of Toxicology, National Taiwan University College of Medicine, Taipei, Taiwan; 4 Center of Faculty Development, National Taiwan University College of Medicine, Taipei, Taiwan; 5 Department of Medical Laboratory, Taoyuan Armed Forces General Hospital, Longtan, Taoyuan 325, Taiwan; 6 Graduate Institute of Medical Science, National Defense Medical Center, Taipei 114, Taiwan; 7 Department of Medical Research, Taipei Tzu Chi Hospital, Buddhist Tzu Chi Medical Foundation, New Taipei City, Taiwan; 8 Department of Medicine, Division of Radiology, Cardinal-Tien Hospital, New Taipei City 231, Taiwan; 9 Graduate Institute of Medical Sciences, College of Medicine, Taipei Medical University, Taipei, Taiwan; 10 Cell Physiology and Molecular Image Research Center, Wan Fang Hospital, Taipei Medical University, Taipei, Taiwan; 11 Anesthesiology and Health Policy Research Center, Taipei Medical University Hospital, Taipei, Taiwan; 12 Department of Medicine, Division of Nephrology, Taipei Tzu Chi Hospital, Buddhist Tzu Chi Medical Foundation, New Taipei City 23142, Taiwan; 13 Department of Medicine, Division of Nephrology, Fu-Jen Catholic University Hospital, School of Medicine, Fu-Jen Catholic University, New Taipei City 24352, Taiwan

**Keywords:** intradialytic cycling, N-terminal telopeptide-1, tartrate-resistant acid phosphatase-5b, osteoporosis, end-stage renal disease, bone turnover

## Abstract

Introduction: Bone turnover markers reflected the bone remodeling process and bone health in clinical studies. Studies on variation of bone remodeling markers in different stage CKD were scant, and this study investigated the role of bedside intradialytic cycling in altering concentrations of bone-remodeling markers in patients with end-stage renal disease (ESRD).

Materials and Methods: Participants were segmented into four groups: a group with eGFR >60 ml/min/1.73 m^2^, a chronic kidney disease group with eGFR 15–60 mL/min/1.73 m^2^), an ESRD group with an exercise intervention, and an ESRD group with standard care. Comparison of bone turnover markers was performed among groups. The intervention consisting of 12 weeks of intradialytic cycling was performed during dialysis. The variation of bone-remodeling markers was compared between the ESRD with exercise along with the ESRD with standard care after 12-week monitoring.

Results: Bone-formative marker levels (bone-specific alkaline phosphatase and procollagen type 1 amino-terminal propeptide, P1NP) were higher in ESRD patients than in non-ESRD patients and were correlated with indoxyl sulfate and intact parathyroid hormone concentrations (*p* < 0.05). Postexercise concentrations of tartrate-resistant acid phosphatase-5b (*p* = 0.003) and N-terminal telopeptide-1 (*p* = 0.001) had increased in the ESRD patients after 12 weeks of bedside cycling. Bone-formative marker concentration was not altered in the exercise group after cycling.

Conclusion: Bone-formative marker concentrations increased with the severity of chronic kidney disease. Bone formative markers concentration increased along with CKD severity. We demonstrated the bone resorptive markers tartrate-resistant acid phosphatase-5b and N-terminal telopeptide-1 increased after intradialytic cycling in ESRD patients.

## INTRODUCTION

Chronic kidney disease (CKD) involves a chronic reduction in the glomerular filtration rate (GFR) or structural deficits in the genitourinary tract. As defined by Kidney Disease: Improving Global Outcomes Organization (KDIGO), CKD is defined as abnormalities of kidney structure or function, present for a minimum of 3 months. The classification of the severity is mainly determined by the glomerular filtration rate and the degree of albuminuria [1]. Imbalances in fluids and electrolyte homeostasis caused by CKD are associated with multiple comorbidities, including cardiovascular disease, renal anemia, congestive heart failure, and CKD–mineral and bone disorder (CKD-MBD). CKD-MBD, which involves renal osteodystrophy, refers to a broad spectrum of pathomorphological bone lesions [2]. CKD-MBD develops as a systemic bone and mineral metabolism disorder and underlies many bone and soft tissue disorders. CKD-MBD comprises three abnormalities: changes in laboratory values, abnormal bone metabolism, and vascular calcification [3]. Bone disorders in CKD patients arise from abnormal mineral metabolism, which decreases bone density and releases uremic toxins that alter bone quality. Nii-Kono et al. observed that uremic toxins hinder parathyroid hormone (PTH)-stimulated intracellular cyclic adenosine monophosphate production, decrease PTH receptor expression, and induce oxidative stress in osteoblast cells which induce adynamic bone disease (ABD) in patients with end-stage renal disease (ESRD) [4]. Furthermore, accumulated uremic toxins adversely affect bone mechanical properties and the chemical composition (i.e., the pentosidine/matrix ratio and the mineral/matrix ratio). Such alterations result in additional deterioration of bone quality and increase the likelihood of hip fracture in CKD patients [5]. Kazama et al. described the resultant bone structural abnormality and microdamage as “uremic osteoporosis” [6]. In the early stages of CKD, uremic osteoporosis causes bone quality loss but not bone quantity loss.

Diagnostic and therapeutic tools for identifying and treating uremic osteoporosis remain unsatisfactory. The treatment of uremic osteoporosis typically involves antiresorptive and anabolic agents. Antiresorptive agents, which include bisphosphonate and monoclonal antibodies against receptor activator of nuclear factor-κB ligand (RANKL)/RANK/osteoprotegerin (OPG), considerably reduce the incidence of hip fractures [7]. PTH analogs, such as teriparatide, increase bone mineral density in vertebrates [8]. However, the incidence of fracture remains high in CKD patients, who typically experience severe post-fracture complications [9]. Lifestyle modifications are vital for managing the progression of various chronic illnesses, including CKD, hypertension, cardiovascular disease, and diabetes mellitus [10]. Aerobic or functional exercise [11] and resistance exercise improved mental health and urea clearance index scores [12]. Physical activity lowers blood pressure and low-density lipoprotein levels by improving cardiac output, stroke volume, heart rate variability, and peak oxygen uptake capacity [13], thereby increasing the body’s sensitivity to insulin and reducing blood sugar levels [14] to weaken the symptoms of CKD. Finally, physical exercise lowers body weight and thus reduces both the burden of adverse conditions caused by obesity and the strain on the kidneys associated with CKD [15].

Bone biopsies are typically performed to assess bone dynamics and bone turnover. However, bone biopsies have several disadvantages and are highly time consuming [16]. An alternative to bone biopsy is the analysis of serum bone-turnover marker levels, which are commonly used to diagnose osteoporosis [17]. Bone-remodeling markers can be categorized as either bone-formative markers or bone-resorption markers depending on whether they originate in osteoblasts or osteoclasts. Osteoclasts release tartrate-resistant acid phosphatase-5b (TRACP-5b) and N-terminal telopeptide-1 (NTX) from osteocytes during bone resorption. By contrast, procollagen type 1 amino-terminal propeptide (P1NP) and bone-specific alkaline phosphatase (ALP) are released from osteoblasts during the bone-formation process [18]. An increased concentration of bone-formative or bone-resorption markers is associated with bone remodeling. Bone-turnover markers have been used to diagnose and treat CKD-MBD. Additionally, the concentrations of bone-turnover markers at multiple stages of CKD have been defined. However, therapeutic interventions for CKD-BMD have observed variations in bone-turnover marker levels [19], and bone formative markers after exercise had been investigated in CKD patients [20]. Thus, peripheral bone-turnover markers may be helpful indicators for the treatment of CKD-MBD.

Our previous study demonstrated that bedside cycling exercise can promote the release of endothelial progenitor cells and bone mineral density in patients with CKD [21]. In addition, such cycling can improve physical function, cardiac function, and respiratory function in CKD patients undergoing dialysis. However, studies regarding bedside cycling and bone-turnover markers, especially the bone resorptive markers, are lacking. The aim of the present study is to investigate the effects of bedside cycling on bone-turnover markers in patients with end-stage renal disease.

## MATERIALS AND METHODS

### Study protocol and ethics

We performed the study at a regional hospital in New Taipei City, Taiwan with adherence to the Declaration of Helsinki. The Ethics Committee for Human Studies of Cardinal Tien Hospital approved the study ethically (CTH-107-3-5-027). The trial registry number was ChiCTR1900025609. This study was performed during August 1st, 2018, to January 31st 2020.

These individuals were recruited via outpatient clinics of nephrology. Participants were provided with informed consent obtained from the trial coordinator. The inclusion criteria were listed as follows: (a) with estimated GFR (eGFR) lower than 60 mL/min/1.73 m^2^ or spot urine proteinuria (diagnosed as urinary protein to creatinine ratio more than 200 mg/gram), (b) older than 20 years old, and (c) with capability for communication verbally in Mandarin Chinese. The exclusion criteria included: (a) recent acute myocardial infarction or unstable angina a within 6 months prior to enrollment; (b) uncontrolled hypertension (more than 190 mmHg in systolic blood pressure); (c) serum hemoglobin lower than 8 g/dL; (d) with active infection cancer, or autoimmune disease; (e) history of emotional instability, a musculoskeletal disability or emotion disorder; (f) uncontrolled cardiac failure, or respiratory problems; (g) history of hospitalization within 1 month prior to enrollment; (h) known bone diseases (e.g., osteomalacia, Paget’s disease); (i) use medications that affect bone metabolism (e.g., bisphosphonates, glucocorticoids) within the past six months; or (j) pregnant or breastfeeding. Upon enrollment, each participant was categorized into one of four groups: (1) group with eGFR >60 mL/min per 1.73 m^2^, (2) CKD group with eGFR = 15–60 mL/min per 1.73 m^2^, (3) an ESRD group receiving an exercise intervention, and (4) an ESRD group with standard care. ESRD was defined as receiving the maintenance hemodialysis (HD) at least more than 3 months continuously. Hemodialysis (HD) was administered three times per week for 3–4 hours each time, with polyether-sulfone used as the dialyzer material.

Pre-study medical assessment for all participants included clinical parameters, baseline hematological and biochemical parameters, baseline dual-energy X-ray absorptiometry (DEXA) measurements, and bone-turnover marker concentrations were collected. For the ESRD patients, bone-turnover markers were evaluated 12 weeks after collection of the first blood sample.

#### Trial design and setting

**Figure 1:**
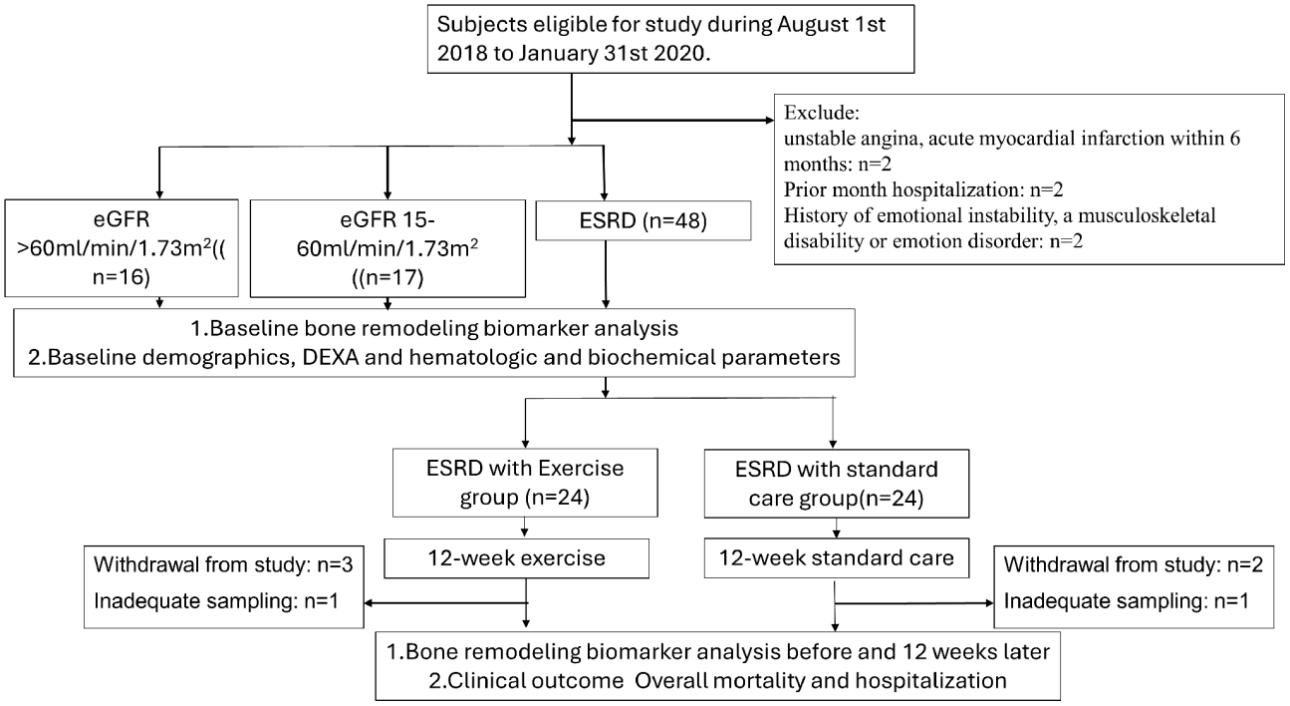
The flow chart of the clinical trial for ESRD participants.

The interventional study for ESRD participants was a non-randomized, opportunistic control, longitudinal design examining the effects of intradialytic exercise in the dialysis center. Figure 1 illustrated the flow chart of the clinical trial.

#### Sample size estimates

We performed the exercise intervention in a single hemodialysis center in northern Taiwan. With the estimate population proportion of 50%, we estimated the sample size as 48 for total participants in ESRD with 95% confidence interval with 8% margin error. The estimated participants in ESRD with and without exercise were 24 individually. Based on the estimated size in ESRD participants, the estimated participant for were 24 for eGFR >60 ml/min and 15~60 ml/min groups.

### Exercise protocol

For the ESRD patients receiving the exercise intervention, a 30-min exercise program was administered during HD treatment in the renal unit over 12 weeks. This program included a 5-min warm-up, 20 min of cycling, and a 5-min cooldown as the protocol from Liao et al. [21]. Briefly, the participants were placed on the bed during the dialysis session, and the pedal trainer exercise bike was placed at the end of bed to facilitate the participants’ position during dialysis session to ensure the comfort and safety during the intervention. Depending on individual conditions, the exercise intervention was initiated 30 to 90 min after basal stabilization. The exercise intervention was performed three times per week, with each session occurring within the first 2 hours of HD. A physician and a nurse specializing in rehabilitation supervised the exercise intervention. Before the intervention, the intensity was checked for every participant in exercise group. The recommended heart rate was defined within 80% of the maximal heart rate. The exercise intensity was started from 8 during initial phase then to 12 to 15 on the Borg Perceived Exertion Scale [22]. Cardiac rhythm was monitored throughout the HD session, and blood pressure was measured every 15 min. Exercise would be terminated if the following symptoms were reported, including chest pain, dizziness, pallor, arrhythmia, hypotension, hypertension or intolerance for exercise. The ESRD patients with standard care received routine medical care.

### Primary and secondary outcomes

We defined the primary outcome as the variation of the bone turnover markers before and 12 weeks after the intervention. The secondary outcome were the overall mortality and hospitalization during 1-year following up duration.

#### Measurement of biochemical laboratory results, indoxyl sulfate, and bone-turnover markers

Within 1 month after the obtainment of written informed consent from the participants, blood sampling would be performed. Blood samples (approximately 10 mL) were collected for all participants. Non fasting blood sampling were performed to avoid discomfort by hypoglycemia. The samples were drawn from a forearm vein while the subjects were seated for non-dialysis participants. Pre-dialytic blood sampling was performed before the dialysis session. After being placed within a vacutainer tube for 30 minutes, the sample would be centrifuged at 1500 × g for 15 minutes at 4°C to separate the serum. The supernatant (serum) was transferred to Eppendorf tubes and stored at −80°C for later analyses of bone formation markers. Other routine parameters, such as hematological and biochemical parameters would be obtained from medical records. Each patient’s estimated eGFR was determined using the Modification of Diet in Renal Disease Study equation [23].

Bone-turnover markers—particularly formation marker with bone-specific ALP (catalog number AC-20F1, IDS Immunodiagnostic Systems, Boldon, United Kingdom), P1NP (number EL-H0185, Elabscience, Wuhan City, China) and resorptive marker with TRACP-5b (catalog number SB-TR201R, IDS Immunodiagnostic Systems, Boldon, United Kingdom), and NTX (Elabscience, Wuhan City, China)—were quantified using enzyme immunoassay kits. For measuring indoxyl sulfate, an enzyme-linked immunosorbent assay kit was applied (Leadgene Biomedical, Tainan, Taiwan) with validation from high-performance liquid chromatography–mass spectrometry (U.S. patent: US10723791B2). Parameters above were detected by enzyme-linked immunosorbent assay method.

These assays were performed per the manufacturer’s instructions, with interassay and intraassay coefficients of variability lower than 9%.

### Aortic calcification measurement based on lateral lumbar X-rays of bone densitometry

Aortic calcification was assessed by analyzing lateral lumbar X-rays following the method outlined in previous studies [24]. A per-segment basis of lumbar vertebral segments L1–L4 serves as the landmark for measuring the extent of calcific deposits. For each segment, a score ranging from 0 to 3 was assigned around the wall of aorta anteriorly and posteriorly. The eight aortic segment scores were then summed to produce a composite abdominal aortic calcification score ranging between 0 and 24.

### Clinical outcome: overall mortality and hospitalization in the ESRD groups

Overall mortality and hospitalization records for the period of April 1, 2018, to December 31, 2021, were systematically compiled by prospectively examining the records of all the patients enrolled in this study for a minimum of 3 months. The cause of death and hospitalization were determined based on all the clinical information available from the Cardinal Tien Hospital. The assessment of hospitalizations included all stays lasting at least one night within a 1-year period following diagnosis. Data for hospitalizations were meticulously gathered from hospital admission records and discharge letters extracted from general practice records.

### Statistics

To demonstrate the continuous variables, the format means ± standard deviations was applied. We presented the categorical variables with format of percentages. The chi-square test was applied to examine the differences in the percentages of categorical variables between groups. The differences in the variables among 4 groups were compared by one-way analysis of variance. A paired *t*-test was conducted to compare pre-exercise and postexercise changes in bone-turnover marker levels in both the ESRD groups. Pearson’s correlation coefficient was used to assess the association between bone-turnover marker levels and individual biochemical and radiologic parameters. We compared the Kaplan–Meier survival estimates for 1-year cardiovascular mortality and hospitalization and between the ESRD groups with and without the exercise intervention. All statistical analyses were performed using SPSS Statistics version 17 for Windows (IBM, Chicago, IL, USA). A two-tailed *p*-value of < 0.05 was considered statistically significant.

## RESULTS

Figure 1 illustrated the flow chart of the study and the participant attrition within study. Under the enrollment phase, 6 subjects were excluded as the exclusion criteria. The participants for eGFR >60 mL/min per 1.73 m^2^, CKD group with eGFR with 15–60 mL/min per 1.73 m^2^ and ESRD group receiving exercise intervention, and ESRD group with standard care were 16, 17, and 48 respectively. The non-random allocation allocated the ESRD with exercise and standard care groups with 24 participants respectively. 3 participants in exercise group withdrew from the study due to intolerability during study. 2 participants in standard care group withdrew from study due to the unwillingness. 2 participants were regarded as inadequate sampling as the result of hemolysis.

### Low bone mineral density and high vascular calcification score in CKD patients

Table 1 presents the patients’ DEXA results and demographic characteristics. The participants in the CKD with eGFR >60 ml/min per 1.73 m^2^ were younger on average than those in the ESRD groups (48.5 ± 2.37 years, vs. 65.64 ± 2.34 years in CKD with eGFR 15~60/min per 1.73 m^2^ ;62.90 ± 3.37 and 58.38 ± 14.95-year-old for ESRD with and without exercise group respectively; *p* < 0.05). In addition, the T-scores were lowest in the eGFR >60 ml/min per 1.73 m (−0.11 ± 0.30, −1.51 ± 0.31 in CKD group; −1.40 ± 0.39 and −1.51 ± 0.26 for ESRD with and without exercise group respectively; *p* < 0.05). The lumbar aortic calcification scores were lowest in the eGFR >60 ml/min per 1.73 m^2^ (0.07 ± 0.06, compared with 2.07 ± 0.63 in the CKD with eGFR 15~59/min per 1.73 m^2^; 7.39 ± 1.41 in the ESRD group receiving the exercise intervention, and 4.71 ± 1.46 in the ESRD group not receiving the exercise intervention). The biochemical results revealed higher concentrations of phosphorus and intact PTH in the CKD and ESRD groups than in the eGFR >60 ml/min per 1.73 m^2^. Regarding bone mineral density and bone composition in the extremities, the upper limb bone mineral density in the left arm was the lowest in the ESRD group not receiving the exercise intervention.

**Table 1:** Baseline demographic characteristics, biochemical laboratory results, and dual-energy X-ray absorptiometry (DEXA) results.

	**GFR >60 ml/min per 1.73 m^2^ (*n* = 16); Mean (SD)**	**GFR 15~60 ml/min per 1.73 m^2^ (*n* = 17); Mean (SD)**	**ESRD with exercise (*n* = 20); Mean (SD)**	**ESRD with standard care (*n* = 21); Mean (SD)**	***P*-value**
Age (years)^a,b,c^	48.5 (2.37)	65.64 (2.34)	62.90 (3.37)	58.38 (14.95)	0.002
Female gender (%)	12 (75)	5 (29.4)	7 (35)	7 (33)	<0.05
Diabetes melitus (%)	1 (6.25)	12 (70)	17 (85)	10 (47.6)	<0.05
Hypertension (%)	3 (18.7)	15 (88.2)	18 (90)	15 (71.4)	<0.05
eGFR (ml/min/m^2^)^a,b,c,d,e^	97.7 (17.64)	35.28 (27.75)	6.43 (1.57)	8.47 (15.77)	<0.05
Hemoglobin (g/dL)^a,b,c,e^	13.17 (1.39)	11.72 (2.19)	11.17 (1.05)	10.71 (1.35)	<0.05
Calcium (mg/dL)^c^	9.61 (0.62)	9.11 (0.70)	9.13 (0.62)	9.04 (0.98)	0.163
Phosphorus (mg/dL)^b,c,e^	3.81 (0.51)	4.22 (0.71)	5.07 (1.50)	5.81 (2.04)	<0.05
Intact parathyroid hormone (pg/mL)^b,c,d^	53.75 (25.82)	157.43 (162.71)	380.06 (358.68)	309.42 (207.85)	<0.05
IL6 (pg/mL)^c,e,f^	3.67 (1.77)	2.99 (1.23)	4.08 (1.08)	17.23 (6.09)	<0.05
Indoxyl sulfate^a,b,c,d,e^	6.54 (3.45)	41.97 (43.96)	202.98 (81.19)	251.61 (98.81)	<0.05
BMC total (g/cm^2^)	2176.36 (79.38)	2078.08 (110.49)	2121.37 (132.13)	2089.78 (82.76)	0.936
BMD total (g/cm^2^)	1.12 (0.02)	1.04 (0.03)	1.05 (0.34)	1.04 (0.11)	0.288
T Score	−0.11 (0.30)	−1.51 (0.31)	−1.40 (0.39)	−1.51 (0.26)	0.053
Z score	−0.07 (0.25)	−0.80 (0.31)	−0.66 (0.31)	−1.15 (0.24)	0.112
Lumbar aortic calcification score^b,c,d^	0.07 (0.06)	2.07 (0.63)	7.39 (1.41)	4.71 (1.46)	<0.05
Left arm total mass (g)	3016.57 (406.68)	3843.35 (246.59)	3855.22 (260.75)	2973.12 (406.79)	0.105
Right arm total mass (g)	4073.92 (240.43)	4074.23 (303.95)	4050.46 (214.80)	4121.93 (234.04)	0.998
Left arm BMC (g)^e,f^	115.80 (16.18)	142.19 (9.35)	151.20 (11.59)	88.30 (14.85)	<0.05
Right arm BMC (g)	158.32 (9.79)	152.18 (11.56)	163.06 (9.01)	146.01 (10.00)	0.651
Left arm BMD (g/cm^2^)^ e,f^	0.59 (0.07)	0.72 (0.02)	0.71 (0.04)	0.48 (0.69)	<0.05
Right arm BMD (g/cm^2^)	0.75 (0.02)	0.71 (0.02)	0.73 (0.02)	0.68 (0.02)	0.277
Left leg total mass (g)	103322.86 (1007.61)	10345.35 (619.00)	9790.83 (455.90)	9917.03 (505.51)	0.891
Right leg total mass (g)	10513.07 (729.63)	10371.35 (601.21)	9880.33 (455.79)	10046.11 (542.50)	0.855
Left leg BMC (g)	401.74 (24.54)	356.34 (26.96)	395.69 (23.39)	330.92 (21.61)	0.171
Right leg BMC (g)	399.34 (23.82)	363.59 (27.30)	395.95 (24.37)	396.21 (284.94)	0.84
Left leg BMD (g/cm^2^)	1.13 (0.04)	1.07 (0.04)	1.18 (0.07)	1.01 (0.03)	0.129
Right leg BMD (g/cm^2^)	1.16 (0.04)	1.08 (0.03)	1.17 (0.05)	1.23 (0.16)	0.657

^a^GFR >60 ml/min per 1.73 vs. GFR 15~60 ml/min per 1.73 m^2^; ^b^GFR >60 ml/min per 1.73 vs. ESRD with exercise; ^c^GFR >60 ml/min per 1.73 vs. ESRD with standard care; ^d^GFR 15~60 ml/min per 1.73 m^2^ vs. ESRD with exercise, ^e^GFR 15~60 ml/min per 1.73 m^2^ vs. ESRD with standard care; ^f^ESRD with exercise vs. ESRD with standard care. Abbreviations: BMD: bone mineral density; BMC: bone mineral content; IL6: interleukin 6.

### Bone-formative marker levels increased with the severity of CKD

Table 2 presents the concentrations of bone-turnover markers. Levels of bone resorption markers, specifically NTX and TRACP-5b, were similar in all the groups. By contrast, levels of bone-formative markers were highest in the ESRD groups, specifically bone-specific ALP (15.57 ± 1.59 pg/mL and 17.24 ± 2.10 pg/mL for the ESRD groups with and without the exercise intervention, respectively) and P1NP (1269.12 ± 116.34 pg/mL and 1634.07 ± 174.57 pg/mL for the ESRD groups with and without the exercise intervention, respectively).

**Table 2:** Baseline concentrations of bone-remodeling markers.

	**eGFR >60 ml/min per 1.73 m^2^ (*n* = 16)**	**eGFR 15~60 ml/min per 1.73 m^2^ CKD (*n* = 17)**	**ESRD with exercise (*n* = 20)**	**ESRD with standard care (*n* = 21)**	***P*-value**
Tartrate-resistant acid phosphatase 5b (U/L)	3.01 (0.21)	3.66 (0.39)	3.91 (0.29)	3.99 (0.32)	0.144
N-terminal telopeptide-1 (ng/ml)	257.62 (27.42)	233.65 (28.09)	182.84 (49.24)	257.32 (27.23)	0.67
Bone specific alkaline phosphatase (µg/L)^a,b^	11.31 (0.87)	11.54 (1.02)	15.57 (1.59)	17.24 (2.10)	<0.05
Procollagen type 1 amino-terminal propeptide (pg/ml)^a,b,c^	697.48 (138.12)	940.68 (126.33)	1269.12 (116.34)	1634.07 (174.57)	<0.05

^a^GFR >60 ml/min per 1.73 vs. ESRD with standard care; ^b^GFR 15~60 ml/min per 1.73 m2 vs. ESRD with standard care. ^c^GFR >60 ml/min per 1.73 vs. ESRD with exercise

### Variations in bone-turnover marker levels in ESRD patients after 12 weeks of exercise

Table 3 presents Pearson correlation coefficient between bone-turnover marker levels and clinical parameters. Concentrations of indoxyl sulfate were positively correlated with concentrations of bone-specific ALP (r = 0.287, *p* < 0.05) and P1NP (r = 0.587, *p* < 0.05). Concentrations of indoxyl sulfate were negatively correlated with concentrations of NTX (r = −0.331, *p* < 0.05). Concentrations of intact PTH were positively correlated with concentrations of bone-specific ALP (r = 0.513, *p* < 0.05) and P1NP (r = 0.341, *p* < 0.05). Finally, the severity of abdominal aortic calcification was positively correlated with concentrations of P1NP (r = 0.305, *p* < 0.05).

**Table 3:** Pearson correlation coefficient of bone-remodeling markers with indoxyl sulfate, parathyroid hormone (PTH) and parameters in DEXA.

	**Tartrate-resistant acid phosphatase 5b**	**N-terminal telopeptide-1**	**Bone specific alkaline phosphatase**	**Procollagen type 1 amino-terminal propeptide**
Indoxyl sulfate	0.177	−0.331^*^	0.287^*^	0.587^*^
iPTH	0.432^*^	−0.142	0.513^*^	0.341^*^
BMC total	−0.128	−0.198	−0.213	0.017
BMD total	−0.135	−0.086	−0.154	−0.012
T Score	−0.175	−0.089	−0.162	−0.053
Z score	−0.158	−0.088	−0.159	−0.034
Lumbar calcification score	0.1	−0.09	0.165	0.307^*^
Left arm BMC	0.082	−0.186	−0.085	0.009
Right arm BMC	0.1	−0.198	−0.027	0.147
Left arm BMD	0.147	−0.127	0.025	−0.022
Right arm BMD	0.113	−0.047	−0.015	0.007
Left leg BMC	0.19	−0.218	−0.006	0.085
Right leg BMC	0.072	−0.067	−0.093	0.093
Left leg BMD	0.128	−0.168	0.04	0.055
Right leg BMD	0.041	−0.036	−0.053	0.082

^*^p < 0.05.

### Bedside cycling induced variations in bone-resorptive-turnover marker levels in patients with ESRD

Table 4 presents variations in the concentrations of bone-resorptive-turnover marker levels before and after the exercise intervention in the ESRD group with the intervention. Specifically, concentrations of TRACP-5b and NTX increased after 12 weeks of exercise in this group (from 3.64 ± 0.25 to 4.23 ± 0.31, *p* < 0.05, and from 113.67 ± 20.53 to 260.61 ± 30.9, *p* < 0.05, respectively). Levels of bone-formative markers were similar before and after the exercise intervention in the relevant ESRD group. Table 5 presents variations in bone-turnover marker levels at the 12-week follow-up in the ESRD group not receiving the exercise intervention. These variations reveal concentrations of bone-resorptive and bone-formative markers similar to those in the ESRD group receiving the exercise intervention.

**Table 4:** Variations in the concentrations of bone-remodeling markers after exercise in patients with ESRD (*n* = 20).

	**Pre exercise Mean (SD)**	**Post exercise Mean (SD)**	***P*-value**
Intact PTH (pg/mL)	380.06 (358.68)	376.59 (236.9)	0.98
Bone formative markers
Procollagen type 1 amino-terminal propeptide (pg/ml)	1433.13 (94.55)	1679.95 (186.74)	0.311
Bone specific alkaline phosphatase (μg/L)	15.3 (1.71)	15.17 (2.11)	0.926
Bone resorptive markers
Tartrate-resistant acid phosphatase 5b (U/L)	3.64 (0.25)	4.23 (0.31)	0.003^*^
N-terminal telopeptide-1 (ng/ml)	113.67 (20.53) 20.53	260.61 (30.9)	0.001^*^

^*^p < 0.05.

**Table 5:** Variations in the concentrations of bone-remodeling markers in patients with ESRD not receiving the exercise intervention (*n* = 21).

	**Pre monitoringMean (SD)**	**Post monitoringMean (SD)**	***P*-value**
Intact PTH (pg/mL)	326.31 (209.6)	310.89 (176.97)	0.825
Bone formative markers
Procollagen type 1 amino-terminal propeptide (pg/ml)	1674.36 (329.35)	1558.68 (393.83)	0.688
Bone specific alkaline phosphatase (μg/L)	17.28 (1.53)	17.56 (1.73)	0.86
Bone resorptive markers
Tartrate-resistant acid phosphatase 5b (U/L)	4.51 (0.46)	4.87 (0.73)	0.484
N-terminal telopeptide-1 (ng/ml)	277.54 (105.45)	322.16 (38.49)	0.589

### Comparison of overall survival and hospitalization rates of ESRD patients after the exercise intervention

**Figure 2:**
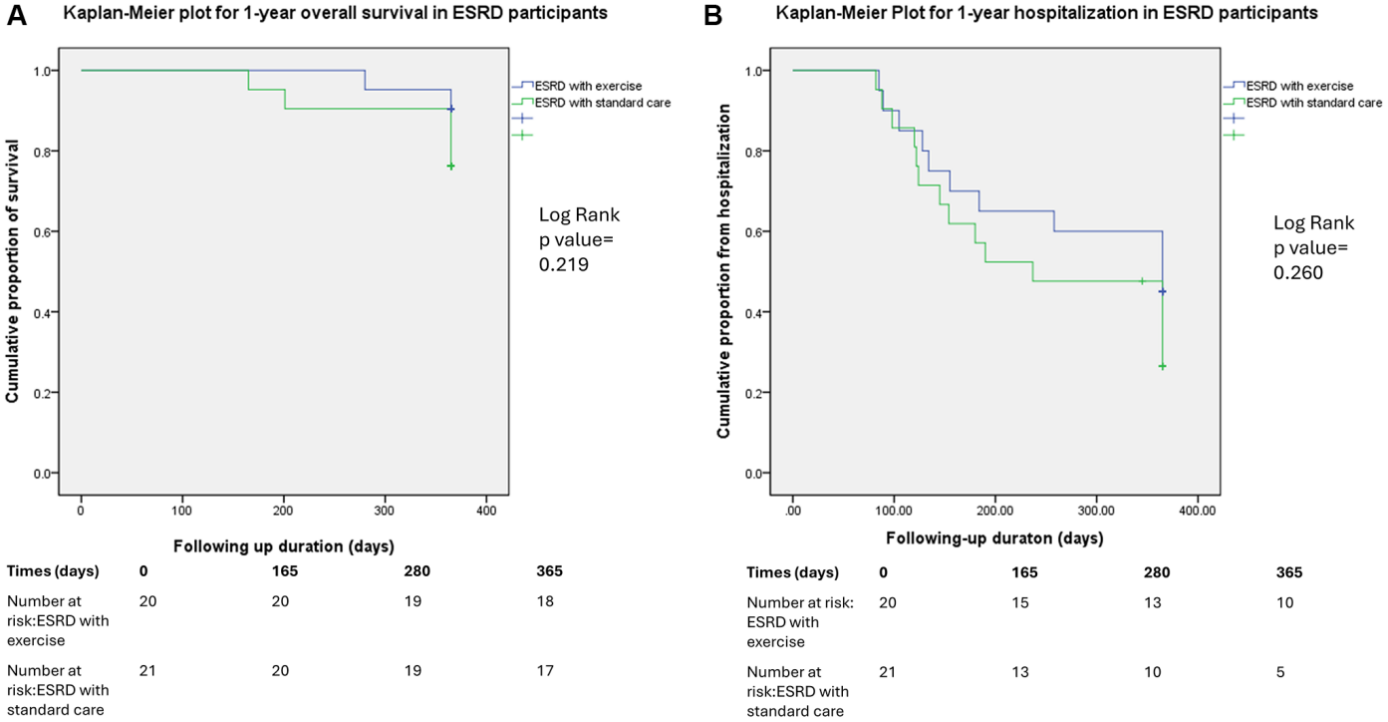
Kaplan–Meier plot comparing the hospitalization (**A**) and overall survival rates (**B**) between the ESRD groups with and without the exercise intervention at the 1-year follow-up.

Figure 2 presents a Kaplan–Meier plot comparing the overall survival (panel A) and hospitalization rates (panel B) between the ESRD groups with and without the exercise intervention at the 1-year follow-up. The cumulative rates of hospitalization and overall survival were similar for both groups.

## DISCUSSION

The present study demonstrated that varying concentrations of bone-turnover markers are present in the different stages of CKD. Specifically, concentrations of bone-formative markers, such as P1NP and bone-specific ALP, increased with the severity of CKD. Additionally, concentrations of indoxyl sulfate and PTH were positively correlated with the concentrations of P1NP and bone-specific ALP. Concentrations of bone-resorptive markers were similar among 4 groups. Indoxyl sulfate and PTH concentrations were differentially correlated with concentrations of NTX and TRACP-5b. In patients with ESRD, 12 weeks of bedside cycling increased the NTX and TRACP-5b concentrations. Finally, the concentrations of all bone-formative markers were similar between the ESRD patients receiving and not receiving the exercise intervention.

In our study, we demonstrated the influence of bone formation marker was similar after intervention. In comparison with previous studies [20, 25–27], the bone formation marker such as bone specific alkaline phosphatase might increase and the PTH hormone should be lowered after intervention. The posture during the dialysis, the combination of resistance exercise and the duration of intervention are the possible factors influencing the outcomes. Our study subjects were lying during the procedure. In the study from Elshinnaway et al., the sitting posture during dialysis was applied [20]. The study by Tabibi et al. used the combination of resistance exercise and then the PTH level could be stabilized [25]. Marinho et al. also showed that the BMD could be improved under the resistance exercise [26]. Mechanical strain is essential for the bone formation [28], and therefore the prolonged lying posture could abate the bone remodeling process and the sequential osteoporosis. The data from Eimori et al. showed the prolonged bedridden status decreases both formative and resorptive markers [29]. Therefore, the combination of resistance might be helpful in enhancing bone formation during dialysis. We used Borg Perceptive Exertion Scale, as the self-reporting scale, to assess the tolerability of the participants. An automatic instrument would be helpful to quantify the resistance. Additionally, CKD severity may affect osteoblast viability. Fukagawa et al. demonstrated that protein-bound uremic toxins suppressed osteoblast function by directly inhibiting cell viability or by exacerbating resistance to PTH [4]. The decreased osteoblast viability might be the reason for the stationary status of bone formative markers.

ALP is a non-specific phosphomonoesterase that processes the hydrolyzing phosphate monoester. Four gene loci encoding the protein moieties of the enzymes determine the isoforms of ALP [30]. Bone-specific ALP originates from the surfaces of osteoblasts. ALP promotes biomineralization by hydrolyzing inorganic pyrophosphates and enforcing extracellular matrix mineralization in osseous tissue [31]. P1NP, a trimeric peptide with a molecular mass of approximately 35,000 kDa, consists of two non-covalently bonded type 1 procollagen-α1 chains and a procollagen-α2 chain. Osteoblasts synthesize the procollagen-1 molecule, and during bone formation, the propeptide extensions at the amino terminals are cleaved off and released into circulation as the collagen molecule is created to produce the osteoid matrix [32]. P1NP levels can thus be used to assess the efficacy of anabolic treatment for osteoporosis [33], and the elevation of serum P1NP levels is a common feature in various pathological conditions associated with enhanced osteoblast viability, including fractures, vitamin D deficiency, and hyperparathyroidism [34]. One study observed that P1NP and bone-specific ALP concentrations increased as the GFR decreased [35]. P1NP clearance depends on the function of the kidneys; therefore, the P1NP concentration is negatively correlated with the GFR [36]. Additionally, the P1NP concentration predicts postmenopausal resorption, whereas P1NP concentration variations are unrelated to bone strength [37]. P1NP levels may also be implicated in bone resorption associated with secondary hyperparathyroidism, which is characterized by an imbalance favoring bone resorption over bone formation. In patients with advanced CKD, phosphaturic PTH is synthesized to compensate for the renal excretion of phosphate. Hyperparathyroidism exacerbates both bone resorption and bone formation. In patients with CKD, bone resorption occurs more frequently than bone formation, leading to a net loss of bone mass. In patients with advanced CKD, vitamin D deficiency and the accumulation of protein-bound uremic toxins (e.g., indoxyl sulfate) reduce the viability of the osteoblast. Impaired osteoblasts are incapable of efficient bone remodeling and increase extraosseous calcium apatite deposition. In conditions with low bone turnover, such as ABD, bone calcium flux buffering is compromised, causing increased calcium exposure and vascular calcification [38]. Consistent with the results of the present study, several studies have demonstrated positive associations between P1NP levels and vascular calcification. Whether this extraosseous calcification involves the generation of type 1 collagen is poorly understood. However, hyperphosphatemia may activate osteoblastic differentiation through the sodium phosphate cotransporter on the vascular smooth muscle cells. Thus, elevated P1NP levels may reflect the severity of extraosseous calcification; the present study supports this association. Nevertheless, additional translational studies are required to clarify the causative associations between aberrant calcification and the expression of type 1 collagen.

In the present study, bone-resorptive marker levels were similar between groups. In dialysis patients, the bone resorptive markers increased after 12 weeks of aerobic intradialytic exercise. To our knowledge, this is the first association between exercise and bone resorptive markers in dialysis patients. TRACP-5b is expressed in osteoclasts and macrophages [39]. An *in vitro* study demonstrated that TRACP-5b concentrations are positively correlated with the number of RANKL-activated osteoclasts [40]. TRACP-5b concentrations have been negatively associated with anti-resorption in multiple clinical investigations [41]. In our earlier study involving individuals with secondary hyperparathyroidism, we observed that active vitamin D reduced levels of both PTH and inflammatory cytokines; additionally, in patients with ESRD, TRACP-5b levels declined following calcitriol treatment and therefore lower the inflammation and oxidative stress [19, 42]. NTX resides in mature type 1 collagen. During the bone resorption process, NTX is released into the circulation; thus, blood NTX levels reflect bone resorption [43]. Additionally, a decrease in NTX levels was observed in a clinical study regarding antiresorptive agents [44]. In one laboratory study, inflammatory cytokines contributed to bone resorption and impeded bone formation [45]. Patients undergoing HD exhibit limited bone turnover, hindered *in vitro* growth, and increased interleukin-6 production in their osteoblasts [46]. One clinical study indicated that inhibiting inflammatory cytokines positively affected bone mineral density [47]. This beneficial outcome is attributable to the dual mechanism of bone-resorption restriction and bone-formation promotion [48]. As the previous discussion, the osteoblast viability was abated under uremic milieu, the activity of osteoclast might be rescued during the aerobic exercise without adequate coupling of osteoblasts [49]. In the present study, the mean PTH values in the ESRD patients receiving and not receiving the exercise intervention were 380.06 ± 358.68 pg/mL and 309.42 ± 207.85 pg/mL, respectively. In addition, mild hyperparathyroidism was observed, even when the PTH levels were within the CKD-MBD parameters set by the Kidney Disease: Improving Global Outcomes organization [50]. The administration of vitamin D or other management for maintaining the viability of osteoblast might be considered as the conjunctive strategy for intradialytic exercise for ESRD patients [51].

For the secondary outcome, the overall mortality and hospitalization within one-year following up duration were similar between the exercise group and standard care group in ESRD subjects. Our previous study demonstrated that bedside cycling promoted the release of endothelial progenitor cells, lowered cytokine levels, and prevented the loss of the femoral neck in a group receiving the same exercise intervention at a 1-year follow-up [21]. Moreover, *in vitro* studies have suggested that endothelial progenitor cells may activate osteogenic differentiation in mesenchymal stem cells and osteoclastic precursor migration [52]. However, contrary to our initial expectations, intradialytic exercise did not alter the concentrations of bone-formative markers in the present study. Several explanations may account for this result: Marinho et al. indicated that an increase in the ALP concentration could be induced by resistance exercise [53]. In the meta-analysis from Ferrari et al. and Sheng et al. [54, 55], the influence on the cardiovascular outcomes and the mortality were neutral for intradialytic exercise. However, the duration of exercise in our study was only 12 weeks. The efficacy for a longer following up duration might not be sufficient, and a larger sample size is required.

This study had several limitations. First, the exercise intervention was limited to 12 weeks, and the long-term effects of exercise on patient health were not evaluated. Second, the exercise intervention was limited to patients with ESRD; thus, the effects of exercise on patients with various stages of CKD were not evaluated. Third, associations between bone-turnover markers and histologic evidence could not be determined, partly because few studies have conducted advanced radiologic investigations to monitor bone remodeling and the viability of osteoblasts. Non-weight-bearing exercises like cycling and swimming do not improve bone mineral density [56] and can lead to variable increases in bone resorption [57]. Factors influencing this variability include exercise control, individual activity history, and tissue-specific adaptations [58]. Bone remodeling is a lifelong process with cycles lasting 4 to 6 months, involving resorption, reversal, and formation of new bone [59]. In chronic kidney disease, bone remodeling is disrupted, increasing fracture risk due to hormonal imbalances and mineral disturbances, complicating uniform remodeling timelines compared to healthy individuals [60, 61]. In end-stage renal disease, uremic toxins cause low bone turnover by inducing oxidative stress, disrupting the balance between bone formation and resorption, leading to reduced bone quantity and quality [62, 63]. Our study observed an increase in bone resorption markers after 12 weeks of cycling exercise. The blood samples taken at this time suggest that cycling may initiate a new phase of bone remodeling, beginning with resorption. These changes in bone markers indicate that cycling exercise could promote beneficial bone turnover, potentially facilitating subsequent bone formation. However, further investigation is needed to clarify this speculation. Bone biopsies performed at multiple time points could provide evidence of exercise-mediated bone remodeling and the benefits of intradialytic exercise in patients with ESRD.

In conclusion, the present study demonstrated that bone-formative marker concentrations increased with a decline in the GFR. Additionally, an increase in the concentration of P1NP was associated with the severity of abdominal aortic calcification. Finally, a cycling exercise intervention improved osteoclastic viability and osteoclast production by elevating serum levels of TRACP-5b and NTX in HD patients with mild secondary hyperparathyroidism.
